# The technical features of the diagnosis or treatment of coronary artery disease through the distal radial artery approach at the anatomical snuffbox compared with the conventional radial artery approach

**DOI:** 10.1186/s13019-022-01979-4

**Published:** 2022-09-07

**Authors:** Yingkai Xu, Hongzhen Niu, Yi Yu, Lixia Yang, Haiyang Wang, Biyang Zhang, Qi Zhao, Qiang Yong, Yujie Zhou

**Affiliations:** 1 Department of Cardiology, Beijing Anzhen Hospital, Beijing Institute of Heart Lung and Blood Vessel Disease, Beijing Key Laboratory of Precision Medicine of Coronary Atherosclerotic Disease, Clinical Center for Coronary Heart Disease, Capital Medical University, 12th Ward, No. 2 Anzhen Road, Chaoyang District, Beijing, 100029 China; 2 Department of Ultrasonography, Beijing Anzhen Hospital, Clinical Center for Coronary Heart Disease, Capital Medical University, Beijing, 100029 China

**Keywords:** Percutaneous coronary intervention, Distal transradial intervention approach, Transradial intervention approach, Radial artery occlusion

## Abstract

**Background:**

To compare the surgical effects of coronary intervention through the transradial intervention (TRI) versus distal transradial intervention (dTRI) approach.

**Methods:**

From September 2020 to April 2021, 302 patients undergoing coronary artery angiography (CAG) or percutaneous coronary intervention in our hospital were retrospectively included. Patients were divided into the TRI group and dTRI group with 151 cases in each group. The technique features, lesion features, and cannulation process were compared between the two groups.

**Results:**

The number of patients who underwent CAG in the dTRI group (35.1%) was significantly greater compared with the TRI group (12.6%) (*P* < 0.01). The rates of triple vessel lesions, calcification lesions and chronic total occlusion lesions were increased in the TRI group compared with the dTRI group (*P* < 0.05). The average radial artery diameter (RAD) in the TRI group (2.550 ± 0.417 mm) was greater than that in the dTRI group (2.070 ± 0.360 mm) (*P* < 0.05). The hemostasis time of the dTRI group (173.272 ± 41.807 min) was lower than that of the TRI group (273.417 ± 42.098 min) (*P* < 0.05). The radial artery occlusion (RAO) rates in the dTRI group (2.6%) were lower than those in the TRI group (8.6%) (*P* < 0.05). The dTRI group had a higher satisfaction score than the TRI group (*P* > 0.05). RAD at the puncture site was a predictor of the overall cannulation success rate with an AUC of 0.747 (95% CI 0.663–0.860; *P* < 0.05).

**Conclusions:**

Despite a steep learning curve, the dTRI approach had a shorter hemostasis time, reduced RAO rates, and notable preliminary safety results compared with the TRI approach. The dTRI approach can be used as a supplemental method to the TRI approach.

## Background

In recent years, the safety and feasibility of percutaneous coronary intervention (PCI) through a transradial approach have been confirmed in numerous large-scale multicenter clinical trials. Transradial coronary intervention (TRI) has become the default approach in PCI and has been widely used and recognized [1, 2]. However, PCI through a transradial approach is associated with a series of comorbidities, including radial artery spasm (RAS), local hematoma, and arteriovenous fistula, with an incidence rate of 1–10% of radial artery occlusion (RAO), which makes the patient lose the opportunity for reintervention through the transradial approach [3, 4]. Therefore, it is crucial to explore new interventional treatment approaches.

PCI or coronary artery angiography (CAG) via distal transradial coronary intervention (dTRI) has become a bright spot in recent years [5]. However, most of these studies are reports of new technical experiences in the initial stage, some of which did not include a control group [6–8]. The detailed types and characteristics of PCI lesions, the characteristics of surgical techniques during coronary intervention, and the safety evaluation of the dTRI approach in the interventional diagnosis and treatment of coronary heart disease remain unknown. Therefore, the purpose of this study was to compare the characteristics of PCI lesions and the surgical effects of coronary intervention through the TRI or dTRI approach and to determine whether dTRI can serve as a supplemental method to the conventional TRI approach.

## Materials and methods

### Study population

From September 2020 to April 2021, 302 consecutive patients undergoing CAG or PCI in Beijing Anzhen Hospital, Capital Medical University were retrospectively included. Patients were divided into the conventional TRI approach group and the dTRI approach group with 151 cases in each group. This study was approved by the ethics committees of Beijing Anzhen Hospital, Capital Medical University (2021082X). All patients signed the consent form.

The inclusion criteria were as follows: (1) patients who conformed to the indications of CAG or PCI; (2) patients with positive Allen’s test results and good collateral circulation of the palm; (3) patients with good and palpable puncture sites of the radial artery and distal radial artery; and (4) patients who agreed to participate in the study. Exclusion criteria included patients (1) with maintenance hemodialysis who had radial artery arteriovenous fistula; (2) with serious tortuous deformation or extreme stenosis of the radial artery or distal radial artery (eg, distal radial artery diameter/radial artery diameter < 1 mm); and (3) who could not tolerate anticoagulation or antiplatelet therapy.

### Color doppler ultrasound

All patients underwent ultrasound examination at the puncture position of the radial artery/distal radial artery with patients in sitting or supine positions preoperatively and 24 h postoperatively. Color Doppler ultrasound equipment (Philips iU 22, Holland) was used to explore the radial artery and distal radial artery. The radial artery exploration vessel segment was at the level of the radial styloid process of the wrist or 1–3 cm above the transverse palmar crease of the wrist, and the distal radial artery exploration vessel segment was the “snuffbox”, a triangular area surrounded by the extensor pollicis longus tendon and the extensor pollicis brevis tendon of the extensor pollicis longus muscle [9]. The diameter and velocity of the radial artery and distal radial artery were measured using color Doppler ultrasound equipment.

### Coronary angiography and intervention procedure

In both groups, all patients were administered 300 mg aspirin (Bayer Ltd, Germany) and 300 mg clopidogrel bisulfate (Sanofi Winthrop Industrie, France) or 180 mg ticagrelor (AstraZeneca, UK) before surgery. Intra-arterial vasodilators drug cocktail (a vasolytic drug cocktail of calcium channel blockers and nitrates) was used in puncture process in two groups to prevent spasm.

For the TRI group, the patients lied supine on the catheter bed after disinfection with the arm straight and abducted and the palm upward to fully expose the puncture site. The level of the radial styloid process of the wrist or 1–3 cm above the transverse palmar crease of the wrist was selected as the puncture site. After adequate local anesthesia with 2% lidocaine, radial artery puncture was performed using Seldinger’s method. Then, a 6 Fr sheath (Terumo, Japan or Cordis, USA) or slender 7Fr sheath (Advanced Polymer and Titanium Medical, China) was chosen by the operator according to the lesion characteristics and operator’s preference. All patients were administered 3000 IU heparin (Tianjin Biochemical Pharmacy, China) intrathecally at the beginning of angiography, and additional heparin was provided during PCI if required (100 IU/kg of body weight). TR BAND® (Terumo Corp., Tokyo, Japan) was used to complete the hemostasis process, and 10 cc (pneumatic) and nonocclusive compression were used to stop bleeding.

For the dTRI group, the patients lied supine on the catheter bed after disinfection, with the arm in a neutral position and the back of the hand outward. Then, the patient was asked to grasp a roll of 4 × 4 gauze in his palm or grasp his thumb under the other four fingers to fully expose the "snuffbox" area. Holding this posture, operators touched the strongest pulsation point of the distal radial artery and then completed the distal radial artery cannulate process with the needle 30–45 degrees to the skin. After the sheath was successfully inserted, operators then completed the coronary intervention procedure using heparin based on the protocol described for in the TRI group. At the end of the operation, the sheath was removed, gauze was folded in half several times to cover the puncture site, and an elastic bandage was used to bypass the thumb with “8-shape” cross compression for fixation. TR BAND radial compression device was used if the band circumference is long enough, otherwise a haemostatic pad of wrapped gauze was used with a firm pressure of elastic bandage for compression [10].

In both groups, the lesion characteristics, stent characteristics, puncture time and hemostasis time of all patients were recorded. The visual analog scale (VAS) pain score was completed by patients before discharge (total possible score is 6 points, 4–6 points indicates severe pain, 2–3 points indicates moderate pain, and 0–1 points indicates slight pain) [11]. The satisfaction questionnaire of the two groups was administered at the time of discharge, and the patients were evaluated according to the degree of pain during the intervention procedure and the comfort of the postoperative hemostasis process. The total possible score was 10 points. Here, 8–10 points denoted satisfaction, 4–7 points denoted basic satisfaction, and less than 4 points indicated dissatisfaction [12]. The criteria of Bleeding Academic Research Consortium (BARC) were used to judge hematoma complications in all patients [13].

### Definitions

Successful cannulation was defined as successful insertion of the radial artery sheath through the TRI or dTRI approach. One-time successful cannulation was defined as successful cannulation through a single puncture via the TRI or dTRI approach. Puncture time was defined as the time from the first puncture to the successful insertion of the radial artery sheath. Procedure success was defined as a successful intervention procedure with a thrombolysis in myocardial infarction (TIMI) blood flow grade of 3 and final residual stenosis < 20% and no deaths, myocardial infarction, emergency bypass or other adverse events before discharge. RAS is defined as the inability to operate catheters or to remove the sheath at the end of the procedure smoothly and painlessly. Radial artery occlusion (RAO) is the absence of a radial pulse and flow signal under Doppler ultrasound. RAO was examined in all patients through color Doppler ultrasound 24 h postoperatively.

### Statistical analysis

All analyses were performed using SPSS 26.0 software (SPSS, Inc., Chicago, IL, USA). Categorical variable values were described using numbers (percentages), and quantitative variables are presented as the mean ± standard deviation (SD). Categorical variables were compared by χ^2^ test or Fisher’s exact test. Normally distributed data were compared using the t test. Potential risk factors for overall cannulation success were investigated first by univariate and multivariate logistic regression models. A ROC curve was used to predict the cutoff. A two-tailed probability value of *P* < 0.05 was considered statistically significant.

## Results

### Baseline and CAG/PCI characteristics

No significant differences in baseline and demographic characteristics, including sex, age, medical history and clinical diagnosis results, were noted between the two groups (*P* > 0.05) (Table 1).

**Table 1:** Baseline information

	dTRI group (n = 151)	TRI group (n = 151)	*P* value
Male, n (%)	107 (70.9)	95 (62.9)	0.142
Age	60.146 ± 9.884	60.397 ± 10.635	0.831
BMI	25.353 ± 2.974	25.963 ± 2.842	0.070
*Medical history, n (%)*			
Hypertension	94 (62.3)	98 (64.9)	0.632
Diabetes mellitus	54 (35.8)	55 (36.4)	0.905
Dyslipidemia	67 (44.4)	60 (39.7)	0.415
Smoking	52 (34.4)	50 (33.1)	0.808
Previous stroke	14 (9.3)	19 (12.6)	0.356
Coronary artery disease	51 (33.8)	47 (31.1)	0.623
Acute coronary syndrome	100 (66.2)	104 (68.9)	0.623
*Clinical diagnosis*			
eGFR	83.681 ± 22.534	82.298 ± 19.883	0.572
LVEF	62.483 ± 6.478	62.029 ± 6.296	0.535
LDL-C (mmol/L)	2.525 ± 1.017	2.603 ± 1.046	0.509

*BMI* body mass index, *eGFR* epidermal growth factor receptor, *LVEF* left ventricular ejection fraction, *LDL-C* low-density lipoprotein cholesterol

The number of patients who underwent CAG in the dTRI group (35.1%) was significantly higher than that in the TRI group (12.6%) (*P* < 0.01). More 7Fr slender sheaths were used in the TRI group (22.5%) compared with the dTRI group (6%) (*P* < 0.01). In the TRI group, the rates of triple vessel lesions, calcification lesions and chronic total occlusion (CTO) lesions were significantly increased compared with those in the dTRI group (*P* < 0.05). The LCX stent diameter in the TRI group (2.942 ± 0.502 mm) was significantly greater than that in the dTRI group (2.659 ± 0.515 mm) (*P* < 0.05). The utilization rate of coronary atherectomy/excimer laser coronary atherectomy (ELCA) in the TRI group (6%) was significantly greater than that in the dTRI group (1.3%) (*P* < 0.05). There were no significant differences in procedure success rates between the two groups (Table 2).

**Table 2:** Intervention features

	dTRI group (n = 151)	TRI group (n = 151)	*P* value
Sheath type			< 0.001
6Fr sheath	142 (94)	117 (77.5)	
7Fr slender sheath	9 (6)	34 (22.5)	
CAG	53 (35.1)	19 (12.6)	< 0.001
PCI and stent features			
Triple vessel lesions	29 (19.2)	46 (30.5)	0.024
Single or double vessel lesions	90 (59.6)	82 (54.3)	0.353
Calcification lesions	14 (9.3)	26 (17.2)	0.042
Restenosis	11 (7.3)	16 (10.6)	0.313
Chronic total occlusion lesions	25 (16.6)	42 (27.8)	0.019
LM lesions	11 (7.3)	16 (10.6)	0.313
LM stent diameter	3.438 ± 0.417	3.469 ± 0.340	0.846
LAD stent diameter	2.917 ± 0.463	3.021 ± 0.351	0.191
LCX stent diameter	2.659 ± 0.515	2.942 ± 0.502	0.019
RCA stent diameter	3.250 ± 0.538	3.125 ± 0.406	0.237
PCI technique			
Coronary atherectomy/ELCA	2 (1.3)	9 (6.0)	0.032
Cutting balloon	20 (13.2)	22 (14.6)	0.739
Drug balloon	32 (21.2)	32 (21.2)	1.000
IVUS*	3 (2.0)	5 (3.3)	0.723
Operation time	52.172 ± 31.026	70.053 ± 41.779	< 0.001
Procedure success	147 (97.4)	146 (96.7)	> 0.999
Contrast volume	88.377 ± 53.039	101.391 ± 54.333	0.036
Total radiation dose	1811.430 ± 2438.773	2871.013 ± 4378.477	0.010

*CAG* coronary angiography, *LM* Left main, *LAD* Left anterior descending artery, *LCX* Left circumflexartery, *RCA* right coronary artery; *PCI* percutaneous transluminal coronary intervention, *ELCA* excimer laser coronary atherectomy, *IVUS* Intravascular ultrasound

*Data was compared by Fisher’s exact test

### Ultrasound examination

As shown in Table 3, the average radial artery diameter (RAD) in the TRI group (2.550 ± 0.417 mm) was significantly greater than that in the dTRI group (2. 070 ± 0.360 mm) (*P* < 0.05). The average RAD of the TRI group was close to the outer diameter of the 6 Fr sheath (2.62 mm, Terumo, Japan), and the RAD of 28.48% of patients in the TRI group was greater than that of the slender 7Fr sheath (2.72 mm, Advanced Polymer and Titanium Medical, China). In the dTRI group, 9.93% of the patients had RADs greater than the outer diameter of the 6 Fr sheath, and 7.28% of patients had an RAD greater than that of the slender 7Fr sheath.

**Table 3:** Ultrasound results

	dTRI group (n = 151)	TRI group (n = 151)	*P* value
RAD in puncture site	2.070 ± 0.360	2.550 ± 0.417	< 0.001
Velocity in puncture site	61.370 ± 16.974	63.793 ± 18.873	0.239

*RAD* radial artery diameter

### Cannulation characteristics

The overall cannulation success rate in TRI group was greater than that in dTRI group (98% vs. 90%) (*P* < 0.05), and the number of puncture attempts required to achieve access in the TRI group were significantly less than that required for the dTRI group (1.139 ± 0.490 vs. 1.656 ± 1.195) (*P* < 0.05), and the one-time successful cannulation rates of TRI group (90.7%) were significantly greater than that of the dTRI group (68.9%) (*P* < 0.05). In the dTRI group, 15 patients failed distal radial artery cannulation, including 6 (4.0%) of whom could not be punctured, 8 (5.3%) of whom could not be wired, and 1 (0.7) who experienced sheath failure. The hemostasis time of the dTRI group was 173.272 ± 41.817 min, which was lower than that of the TRI group (273.417 ± 42.098 min) (*P* < 0.05), but the puncture time of the dTRI group was greater than that of the TRI group (3.479 ± 1.102 vs. 1.207 ± 0.659) (*P* < 0.05). The VAS pain score was significantly higher in the dTRI group compared with the TRI group (*P* < 0.05). Although not significantly different, the dTRI group had a higher satisfaction score than the TRI group (*P* > 0.05) (Table 4).

**Table 4:** Cannulation characteristics

	dTRI group (n = 151)	TRI group (n = 151)	*P* value
Whole success cannulation	136 (90.0)	148 (98.0)	0.04
Number of puncture attempts	1.656 ± 1.195	1.139 ± 0.490	< 0.001
One-time successful cannulation	104 (68.9)	137 (90.7)	< 0.001
Crossover to other approach			
Cannot puncture*	6 (4.0)	1 (0.7)	0.121
Cannot wire*	8 (5.3)	0	0.007
Sheath failure*	1 (0.7)	2 (1.3)	> 0.999
Puncture time (min)	3.479 ± 1.102	1.207 ± 0.659	< 0.001
Hemostasis time (min)	173.272 ± 41.817	273.417 ± 42.098	< 0.001
VAS pain score	2.311 ± 3.858	1.543 ± 0.839	< 0.01
Satisfaction score	8.146 ± 1.157	7.940 ± 1.555	0.194

*VAS* visual analogue scale

*Data was compared by Fisher’s exact test

### Postoperative complications

The radial artery occlusion (RAO) rates in the dTRI group (2.6%) were significantly lower than those in the TRI group (8.6%) (Fig. 3) (*P* < 0.05), but the spasm rates of the dTRI group in the radial artery were greater than those of the TRI group (9.3% vs. 2.6%) (*P* < 0.05). No significant differences in puncture site hematoma, numbness, arteriovenous fistula or other complications were noted between the two groups (Table 5).

**Table 5:** Radial artery complications

	dTRI group (n = 151)	TRI group (n = 151)	*P* value
RAO	4 (2.6)	13 (8.6)	0.025
Spasm	14 (9.3)	4 (2.6)	0.015
Dissection*	1 (0.7)	2 (1.3)	> 0.999
BARC Grade			
0	143 (94.7)	133 (88.1)	0.040
1–2	8 (5.3)	17 (11.3)	0.060
3–5*	0	1 (0.7)	1.000
Numbness*	2 (1.3)	4 (2.6)	0.684
Arteriovenous fistula*	1 (0.7)	3 (2.0)	0.622

*RAO* radial artery occlusion, *BARC* bleeding academic research consortium

*Data was compared by Fisher’s exact test

### Predictors of overall cannulation success rate in the dTRI group

**Fig. 1:**
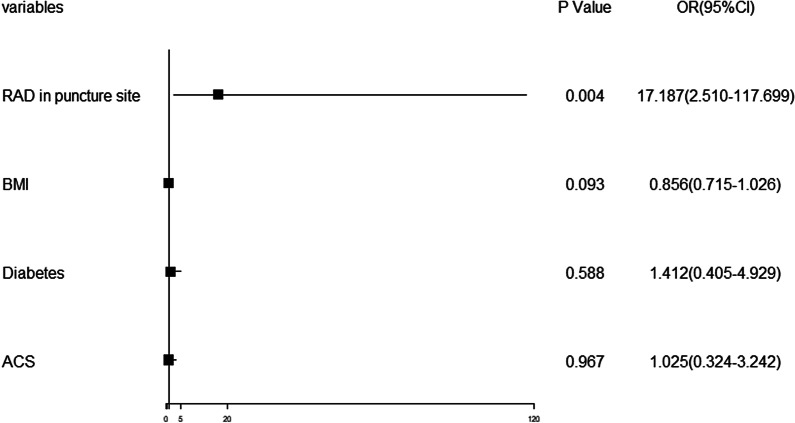
Multivariate analysis of the overall cannulation success rate in the dTRI group

**Fig. 2:**
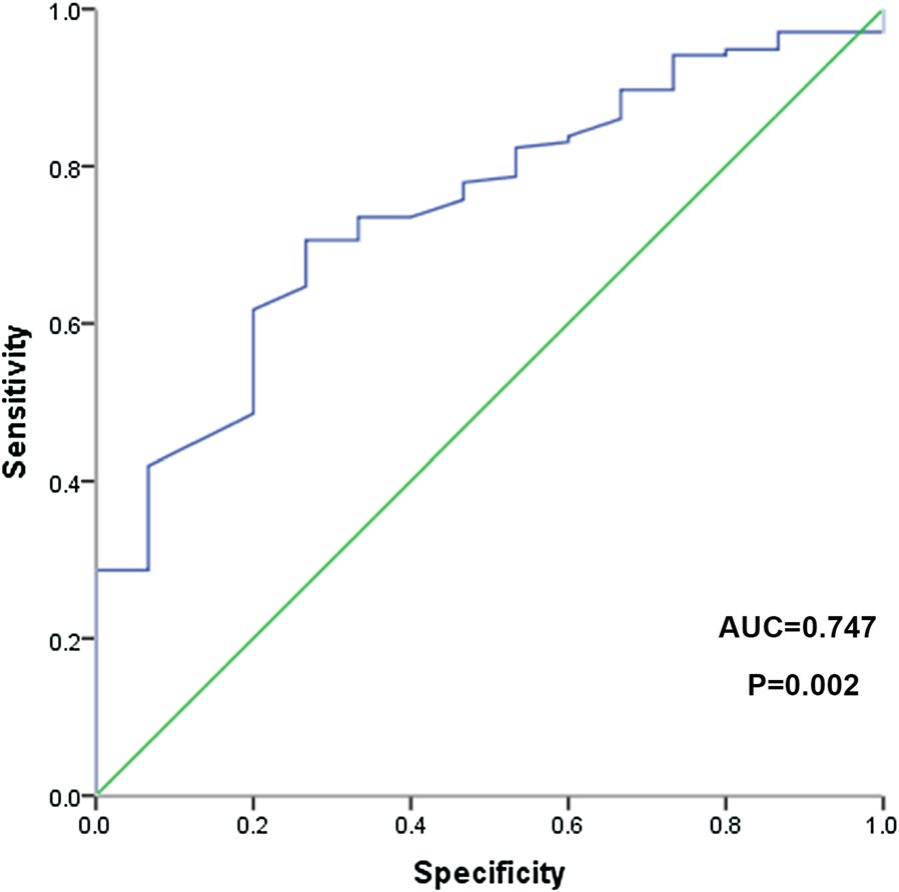
ROC curve of RAD in the puncture site for the overall cannulation success rate in the dTRI group. The AUC was 0.747 (95% CI 0.663–0.860, *P* = 0.002). The cutoff was 1.955 mm with a specificity of 70.6% and sensitivity of 73.30%

The effects of sex, age, BMI, hypertension, diabetes mellitus, dyslipidemia, smoking, LDL-C, acute coronary syndrome and RAD at the puncture site on the overall cannulation success rate in the dTRI group were further analyzed. Logistic multivariate analysis results showed that RAD at the puncture site was a predictor of the overall cannulation success rate with an AUC of 0.747 (95% CI 0.663–0.860; *P* = 0.002) (Table 6, Figs. 1 and 2).

**Table 6:** Univariate analysis of whole success cannulation rate in dTRI group

	OR	95%CI	*P* value
Male	0.873	0.262–2.904	0.824
Age	0.980	0.928–1.036	0.477
BMI	0.896	0.752–1.068	0.219
Hypertension	1.505	0.515–4.399	0.455
Diabetes mellitus	1.599	0.483–5.289	0.442
Dyslipidemia	0.670	0.230–1.954	0.464
Smoking	1.056	0.341–3.270	0.924
LDL-C	0.854	0.513–1.420	0.543
Acute coronary syndrome	1.348	0.452–4.022	0.592
RAD in puncture site	13.243	2.203–79.621	0.005

*LDL-C* low-density lipoprotein cholesterol, *RAD* radial artery diameter

## Discussion

**Fig. 3:**
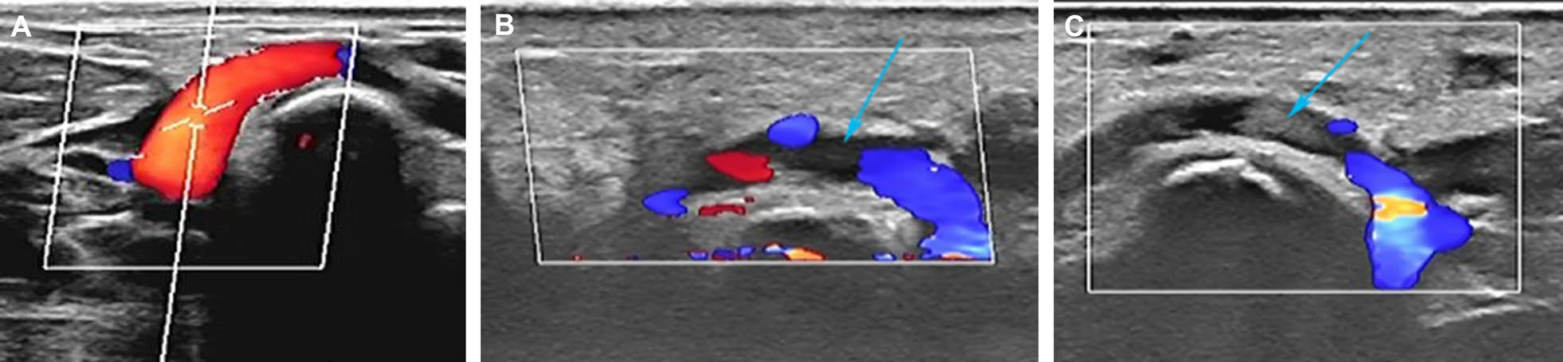
Color Doppler ultrasound images. **A** Normal distal radial artery. **B**. Distal radial artery occlusion and loss of blood flow signals (blue arrow). **C** Distal radial artery occlusion and occluded hypoechoic mass (blue arrow)

The distal radial artery is formed by the dorsal carpal branch of the proximal radial artery, which forms the deep palmar arch with the branches of the ulnar artery. Operators can palpate the distal radial artery in the "snuffbox" area, offering a new approach for the interventional treatment of coronary heart disease [14]. The average diameter of the distal radial artery was 2.04 ± 0.43 mm in males and 1.96 ± 0.44 mm in females, meeting the basic requirements of the interventional approach [15]. As a new interventional approach, the distal radial artery can achieve a lower radial artery occlusion rate, which has been confirmed in some studies [6–8, 16]. However, the operation and lesion characteristics of coronary angiography and interventional therapy through the distal radial artery are generally unknown (Fig. 3).

In our study, 53 (35.1%) patients underwent CAG, and 98 (64.9%) patients underwent PCI in the dTRI group. These values were was similar to those reported in a study by Yu et al. [17]. The TRI group had higher rates of triple vessel lesions, calcification lesions, CTO lesions, and coronary atherectomy/ELCA techniques compared with the dTRI group. The possible reasons are as follows. For complex lesions, a slender 7Fr sheath combined with 7Fr guidance can provide a higher supporting force to successfully meet the needs of complex operations, such as coronary rotational atherectomy, and to completely address a series of complex lesions, such as CTO and calcification. The RAD of the distal radial artery is relatively small, and the use of a large-diameter sheath may aggravate damage to the radial artery intima and media. On the other hand, some interventional devices (such as guiding, laser catheter, etc.) may not possess sufficient length to treat some patients with long arm and tall stature given that the distal radial artery approach is closer to the distal end of the heart. The LCX stent diameter in the TRI group was greater than that in the dTRI group. The conventional radial artery approach has more advantages in the treatment of lesions with larger reference vessel diameters. During the intervention process, the guiding catheter does not need to be passed through the dorsal wrist to the proximal radial artery, which facilitates the delivery of a larger stent and balloon.

The procedure success rate of 97.4% in the dTRI group was consistent with other studies [17, 18]. Therefore, the distal radial artery approach can complete coronary angiography and most PCI processes with a high procedure success rate and good immediate angiography results. The operation time, contrast volume and total radiation dose in the dTRI group were lower than those in the TRI group but were greater than those in Kim Y and Valsecchi O’s study [19, 20]. Various possible reasons can explain the differences. For example, the surgical complexity of all patients in this study was greater than that of the above two studies, and the number of CTOs, triple vessel lesions and other complex lesions and techniques used in the TRI group was greater than that in the dTRI group.

Preoperative radial artery examination of the puncture site is helpful and necessary to determine the vascular condition (whether there is tortuosity or occlusion) in the radial artery and improve the success rate of the puncture process. The average RAD of the TRI group was greater than that of the dTRI group, which is similar to Meo D’s conclusion and consistent with the anatomical structure [21]. The overall cannulation success rate in the dTRI group was lower than that in the TRI group, and the dTRI group had more puncture attempts and lower one-time successful cannulation rates, which were consistent with the results of Coomes EA’s study [22]. The learning curve of the new puncture technique, the difficulty in palpating the distal radial artery, and the relatively small diameter of the distal radial artery may explain the result.

Six (4.0%) cannulation failures were puncture failures, 8 (5.3%) were wire failures, and 1 (0.7%) was attributed to sheath failure in the dTRI group. The relative tortuosity of the distal radial artery compared with the conventional radial artery may explain the wire failure, whereas the small RAD of the distal radial artery may explain puncture failure. The compression time (273.417 ± 42.098 min) of the TRI group in this study was similar to that of a Japanese study (378 ± 253 min) [23], reporting that the compression time was short in Europe and the United States and long in Asia. This finding may be related to the slower metabolism of heparin in Asians [24]. The hemostasis time of the dTRI group (173.272 ± 41.817 min) was less than that of the TRI group, which was similar to the findings of Vefalı V’s study given the superficial location and the relatively small RAD of the distal radial artery [25]. Therefore, it was easier to compress and finish the hemostasis procedure. This may also explain the finding that although the VAS pain score in the dTRI group was greater than that in the TRI group, the satisfaction score of the two groups was not significantly different, which was similar to that reported in Lee JW’s study [12]. In contrast to those patients in the TRI group, patients under hemostasis in the dTRI group could still move the wrist in an unconstrained fashion; thus, these patients’ quality of life was not affected.

The RAO rates of the dTRI group were lower than those of the TRI group, which was consistent with the conclusion of Cai et al. [26]. This finding may be attributed to the double blood supply of the distal radial artery formed by the superficial palmar arch and deep palmar arch. The smaller RAD of the dTRI group may explain the increased incidence of RAS in the dTRI group. No significant differences in serious complications, including severe hematoma and arteriovenous fistula, were observed between the groups, which was similar to the research results of Lee JW et al. [12].

RAD at the puncture site and other indicators, including BMI, diabetes and acute coronary syndrome (ACS) as described by our clinical experience and a previous study [27], were included in multivariate analysis of the overall cannulation rate success rate in the dTRI group. We found that RAD with an AUC of 0.747 (95% CI 0.663–0.860) was a predictor for the overall cannulation success rate, which may be explained by the notion that a larger diameter can enhance palpation of the distal radial artery. BMI was identified as a risk factor for cannulation failure in a previous study [27]. This inconsistency may be due to the different sample sizes.

Some limitations in this study should be noted. The effect of coronary intervention via the dTRI approach on the treatment of complex and high-risk lesions and the effect on complex coronary intervention techniques via the dTRI approach were not determined. In the future, the exact indications, safety, and feasibility of distal radial artery interventional therapy should be confirmed by further large-scale, multicenter clinical trials. In addition, the distal transradial approach refers to two distinct entry sites, including the anatomical snuffbox and dorsum of the hand. The dorsum of the hand approach was not studied, and we will consider assessing the use of this approach in future studies [28].

## Conclusion

In conclusion, the distal radial artery approach exhibits good immediate effects in the completion of CAG and noncomplex coronary intervention. The dTRI approach can be used as an option for interventional physicians in the treatment of most noncomplex lesions that do not require numerous complicated techniques given that the dTRI approach has a good immediate effect in the completion of CAG and noncomplex coronary intervention as well as a shorter hemostasis time, lower RAO rates, and preliminary safety and feasibility than the TRI approach. In addition, if larger catheters are needed in the PCI procedure, a thin-wall/7 Fr slender sheath can reduce blood vessel wall damage and achieve good clinical angiography effects. Thus, some complex lesions can be addressed using these techniques [28, 29].

## Data Availability

All data generated or analyzed during this study are included in this published article.

## Abbreviations

TRI: Transradial intervention
dTRI: Distal transradial intervention
CAG: Coronary artery angiography
PCI: Percutaneous coronary intervention
RAD: Radial artery diameter
RAO: Radial artery occlusion
CAG: Oronary artery angiography
CABG: Coronary artery bypass grafting
VAS: Visual analogue scale
TIMI: Thrombolysis in myocardial infarction
RAS: Radial artery spasm
